# Promoting mucosal healing by targeting TMEM219-dependent intestinal epithelial stem cell defects in inflammatory bowel disease

**DOI:** 10.1172/JCI192640

**Published:** 2025-05-15

**Authors:** Nicolas Schlegel

**Affiliations:** Department of General, Visceral, Transplant, Vascular and Pediatric Surgery, University Hospital Würzburg, Oberduerrbacherstrasse 6, Wuerzburg, Germany.

## Abstract

Inflammatory Bowel Diseases (IBD), including Crohn’s disease and ulcerative colitis, pose challenges due to their complex pathophysiology and high prevalence. Despite advances in immune-targeted therapies, a substantial number of patients fail to achieve mucosal healing, highlighting the need for alternative therapeutic strategies. In this issue of the *JCI*, D’Addio et al. identified another mechanism underlying impaired epithelial regeneration in Crohn’s disease. They found that abnormal cell death in intestinal epithelial stem cells, mediated by altered TMEM219 signaling, led to impaired mucosal healing. Targeting TMEM219 with ecto-TMEM219, which blocks its activation, restored stem cell function and promoted mucosal healing in vitro and in vivo. These findings suggest a promising therapeutic avenue focusing on epithelial repair. Additionally, patient-derived organoids (PDOs) emerge as a valuable tool for personalized treatment strategies and for advancing the field of IBD research. This study underscores the importance of epithelial cell biology in developing innovative IBD therapies.

## Mucosal healing as a treatment goal

Inflammatory Bowel Diseases (IBD) represent a major socioeconomic burden due to a rising incidence and prevalence, making IBD a challenging task for healthcare systems worldwide (1, 2). The pathophysiology of IBD, encompassing Crohn’s disease, ulcerative colitis, indeterminate colitis, and additional subtypes, remains incompletely understood (3). It is known that IBD is caused by multiple factors including genetic predisposition, aberrant immune reactions, changes of the microbiota, environmental aspects, and epithelial defects (4). Crohn’s disease and ulcerative colitis show distinctions concerning their molecular, immunological, and clinical phenotypes, but both types share epithelial dysfunction as a common feature resulting in compromised barrier function and reduced regenerative capacity of the mucosa (5). The importance of repairing inflammation-induced epithelial lesions to restore epithelial integrity has led to the concept termed ‘‘mucosal healing,’’ which has become the standard treatment goal of IBD (6–8).

In the last decades, the focus of IBD research has been linked with a better understanding of aberrant immune responses. Currently, virtually all medical IBD therapies target the immune system. However, it has been reported that 40%–70% of patients do not show mucosal healing or endoscopic improvement, despite receiving immune-targeted therapies, which underscores the need for innovative therapeutic approaches targeting alternative cell types or mechanisms (4, 5, 9). Translational research contributing to our understanding of the cellular mechanisms underlying impaired epithelial regeneration and mucosal healing will prime future therapeutic approaches for patients with IBD (10, 11).

## Targeting intestinal epithelial stem cell defects

It has been suggested that intestinal stem cell defects contribute to delayed mucosal healing in IBD, which has been attributed to increased cell death. The precise reason why so many patients with IBD show defects in mucosal healing has been largely unclear. Some studies have shown Caspase 8 and increased expression levels of receptor-interacting protein3 (RIP 3) as being involved in intestinal epithelial cell death and intestinal inflammation (12, 13).

In this issue of the *JCI*, the work of D’Addio and coworkers sheds new light on this important pathophysiological aspect for patients with Crohn’s disease (14). They report abnormal cell death in intestinal epithelial stem cells, which leads to an exacerbation of colitis and limits mucosal regeneration. The cause for the increased death in intestinal epithelial cells was linked to changes in the expression of a cell death factor named transmembrane 219 (TMEM219) (14). This protein has previously been reported as a regulator of beta cells and as a cell death receptor in breast and prostate cancers (15, 16) and is activated by its ligand, insulin-like growth factor binding protein 3 (IGFBP3) (15). D’Addio and coworkers showed that TMEM219 expression was altered. Moreover, in a cohort of patients with a refractory course of Crohn’s disease or active Crohn’s disease, TMEM219 signaling was activated and correlated with a failure of mucosal regeneration (14).

**Figure 1:**
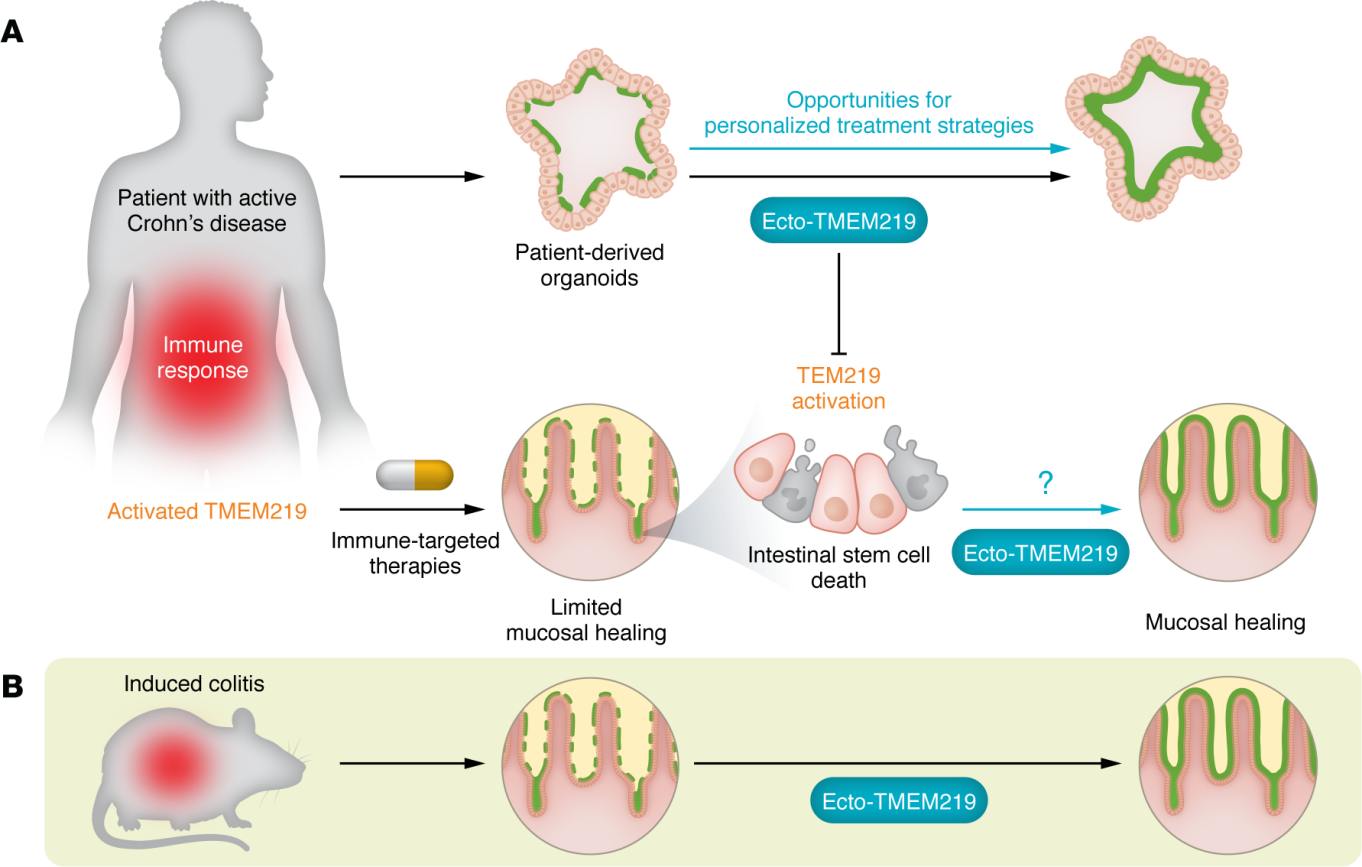
Targeting TMEM219-dependent intestinal epithelial stem cell defects promotes mucosal healing. (**A**) Patients with refractory or active Crohn’s disease showed overactive TMEM219 signaling that correlated with a failure to regenerate the mucus layer, despite immune suppression. TMEM219 contributed to cell death in intestinal epithelial stem cells. In mini-guts derived from patients with Crohn’s disease, targeting the TMEM219 axis using ecto-TMEM219 restored self renewal. Beyond standard immune-targeted treatments, clinically targeting intestinal epithelial cells using TMEM219 blockade may promote mucosal healing. Moreover, PDOs provide a personalized-screening platform for identifying compounds that optimize mucosal restoration. (**B**) ecto-TMEM219 also ameliorated mucosal healing in DSS-induced and T cell–mediated colitis models.

As a proof of concept, the authors used recombinant protein ecto-TMEM219, which attenuates IGFBP3/TMEM219 binding and signaling (14). Targeting TMEM219 using ecto-TMEM219 restored the self-renewal abilities of mini-guts generated from patients with Crohn’s disease in vitro. Furthermore, ecto-TMEM219 ameliorated DSS-induced and T cell–mediated colitis in vivo, ultimately leading to mucosal healing. This finding represents a major step toward better understanding defects of mucosal healing from a mechanistic point of view. In addition, this treatment strategy could be part of the long-awaited approach for targeting intestinal epithelial stem cell defects in order to overcome delayed mucosal healing (Figure 1).

## A strategy for improving patient outcomes

Apart from targeting IGFBP3/TMEM219, the use of PDOs provides an opportunity to assess alternative therapeutic options for patients with IBD and to increase our understanding of a primary role of intestinal epithelial cells or intestinal epithelial stem cells in the pathophysiology of IBD. The PDO approach has already been established for patients with colorectal cancer, where PDOs have been used to predict individual therapeutic response to medications (17, 18). This method allows for the selection of therapies most likely to improve the outcome for patients with cancer. For IBD, this approach has also been suggested before. However, an important argument against the use of PDOs from patients with IBD posits that such an approach would ignore the contribution of immune cells to the onset and perpetuation of the disease. This argument overlooks evidence from several groups, including the current work of D’Addio et al., showing that organoids from patients with IBD maintain typical features of the disease (14, 19–21), even in the absence of proinflammatory mediators and immune cells. The current observation of an intrinsic upregulation of the cell death factor in intestinal stem cells from patients with Crohn’s disease supports this notion. This observation may also explain, to some extent, how organoid cultures from patients with IBD that are derived from sites of severe inflammation are more difficult to cultivate than organoids from healthy individuals. Overall, these considerations and findings support the idea that PDOs involving IBD will be a useful tool to search for individual therapeutic agents that promote mucosal healing and intestinal epithelial barrier restoration (5).

In summary, research to understand intestinal epithelial cell turnover, differentiation leading to barrier maturation, and mucosal healing still remains underrepresented in IBD research. We applaud D’Addio and colleagues for making an important step towards understanding the pathophysiology of intestinal epithelial stem cell defects.

## Version 1 05/15/2025

Electronic publication
